# Identity Pathology and Emptiness as Novel Predictors of Suicidal Ideation

**DOI:** 10.1111/sltb.13164

**Published:** 2025-01-18

**Authors:** Brianna Meddaoui, Jeremy G. Stewart, Erin A. Kaufman

**Affiliations:** ^1^ Department of Psychology University of Western Ontario London Ontario Canada; ^2^ Department of Psychology Queen's University Kingston Ontario Canada; ^3^ Department of Psychiatry University of Utah Salt Lake City Utah USA

**Keywords:** emptiness, identity pathology, suicide

## Abstract

Emptiness and identity pathology are understudied clinical constructs that overlap, co‐occur, and predict suicidal ideation (SI). However, specific risk pathways have yet to be formally tested.

**Aim:**

We examined whether identity pathology was indirectly associated with future SI via emptiness, and tested impulsivity and emotion dysregulation as moderators.

**Methods:**

Participants (*N* = 251) completed baseline questionnaires assessing SI, borderline personality disorder symptoms, emotion dysregulation, and impulsivity, and SI 2 months later.

**Results:**

Identity pathology was indirectly associated with future SI via emptiness, controlling for baseline SI (*β* = 0.15, Bootstrap 95% CI = [0.06, 0.24]). There was a two‐way interaction between emptiness and both poor use of emotion regulation strategies (*β* = 0.06, *p* < 0.001) and impulsive lack of premeditation (*β* = 0.09, *p* = 0.03) predicting SI.

**Conclusion:**

Those with greater identity pathology were more likely to experience emptiness, which was in turn associated with future SI. Participants who felt empty were also more likely to experience SI when they also reported an inability to use emotion regulation strategies and a tendency to act without considering the consequences. We provide preliminary support for an untested risk pathway for SI, highlighting the need to further study these important experiences.

Suicidal ideation (SI) is distressing and prevalent (Ivey‐Stephenson et al. 2022). Accurately predicting suicidal thoughts and behaviors (STBs) remains challenging, which stymies prevention efforts (Franklin et al. 2017). Meta‐analytic work has identified two key areas of need: investigating novel risk factors and formally testing suicide theories (Franklin et al. 2017). Indeed, most research has repeatedly examined common correlates (e.g., demographics) to the detriment of investigating novel, specific risk pathways. *Emptiness* and *identity pathology* are two understudied constructs that may contribute to our understanding of SI. Both experiences occur transdiagnostically (Rhodes, Hackney, and Smith 2019; Zandersen and Parnas 2019) and are elevated among persons with borderline personality disorder (BPD), a diagnostic group wherein SI is common (Black et al. 2004).

Identity pathology refers to distress and/or confusion about one's sense of self and a lack of continuity in one's beliefs, values, behaviors, and self‐image across contexts or time (American Psychiatric Association [APA] 2013; Kaufman, Montgomery, and Crowell 2014; Westen, Betan, and DeFife 2011). Such pathology can include a perception that one lacks an identity altogether (Kaufman, Cundiff, and Crowell 2015). Emptiness is the subjective absence of emotion and meaning, which typically causes distress and leaves individuals feeling hollow, defective, and alone (Price et al. 2019; Miller et al. 2020). Though relatively understudied, both experiences are associated with distress and impairment in several domains of functioning (Ellison et al. 2016; Morgan et al. 2013; Ren et al. 2018).

Identity pathology and emptiness may each be relevant to understanding suicide risk. Identity pathology is associated with SI and suicide attempts (Ellison et al. 2016), controlling for symptoms of depression (Sekowski et al. 2022), and negative life events (Sokol and Eisenheim 2016). This is perhaps unsurprising, as identity pathology undermines one's sense of purpose—an established protective factor for STB (Kleiman and Beaver 2013). Emptiness is also associated with STB over and above other BPD symptoms that predict SI (e.g., relationship instability; Ellison et al. 2016; Grilo and Udo 2021; Klonsky 2008). In qualitative studies, emptiness is a common theme among suicide attempt survivors (Segal‐Engelchin et al. 2015; Simões, Dos Santos, and Martinho 2020) and in post‐mortem research examining suicide notes (Chia, Chia, and Tai 2008). Identity pathology and emptiness also complement extant suicide theories. Baumeister (1990) suggests suicide is an escape from *painful self‐awareness*, which results from a poor self‐image and sense of purpose. He conceptualizes suicide as an escape from one's distressing immediate context, unbearable emotional pain, and even oneself (Baumeister 1990). He and others have described a person's identity and emotional experiences as essential for developing a sense of purpose and well‐being (e.g., Westen, Betan, and DeFife 2011). Thus, there are already conceptual links between identity pathology, emptiness, and the desire to escape distress via suicide. Although these relations have not been rigorously tested, existing work may suggest a common pathway by which both emptiness and identity pathology elevate the risk of SI.

Identity pathology and emptiness often co‐occur and overlap to some degree. Kernberg's (1967) object relations theory and Linehan's (1993) biosocial model each propose that emptiness is reflective of self‐dysregulation and disrupted identity. Specific etiological pathways have not been investigated; however, it is likely that both share common underlying vulnerabilities and are reciprocally reinforcing (Linehan 1993). Identity provides essential information about how persons view themselves in their broader context, relate to others, and behave in consistent, self‐defining ways (Kaufman and Crowell 2018; Marcia 1989). Those who struggle with their identity typically feel disconnected from themselves and others, which may limit their capacity to perceive, identify, or fully experience their emotions (Rebok et al. 2015; Taylor and Goritsas 1994). Likewise, feeling empty may introduce or maintain identity confusion and interfere with one's self‐expression (Linehan 1993). Prior studies support this hypothesis, such that identity pathology and emptiness are correlated, described similarly, and can be treated concurrently (Ntshingila et al. 2016; Price et al. 2020; Black, Blum, and Allen 2018).

Though distinct, identity pathology and emptiness are both indicative of broader difficulties with emotional and behavioral regulation (D'Agostino et al. 2020; Kaufman and Crowell 2018). Understanding, accepting, and modulating emotions and behaviors is a precursor to healthy identity development (Crocetti et al. 2022; Shtiwi 2023). In particular, research indicates that emotion dysregulation is associated with identity pathology in clinical and non‐clinical samples, and this effect remains after controlling for BPD and depressive symptoms (Biberdzic et al. 2021; Neacsiu et al. 2015). Likewise, emptiness may reflect an attempt to inhibit intense emotions, thereby functioning as a maladaptive method of emotion regulation (Linehan 1993). Indeed, emptiness is moderately correlated with overall trait‐level emotion dysregulation and impulsivity across studies (Ellison et al. 2016; Ermis‐Demirtas et al. 2022; Price, Mahler, and Hopwood 2022). Theoretically, feeling empty represents an ideal condition for impulsivity to manifest, as individuals may attempt to *feel something* by engaging in risky or maladaptive behaviors (Linehan 1993). This aligns with evidence that emptiness indirectly predicts poor work outcomes via impulsivity, which suggests that emptiness and impulse control difficulties synergistically beget harm (Miller et al. 2018). Researchers also hypothesize that STBs may be a method of resolving or tolerating the distress that accompanies emptiness (Yen et al. 2021). Thus, it is possible that STBs emerge as a response to emptiness in some individuals, and this risk pathway is in part influenced by susceptibility to impulsivity and emotion dysregulation.

Emotion dysregulation and impulsivity are among the most widely studied risk factors that contribute to STB (e.g., Brausch and Woods 2019; Bruno et al. 2023). Difficulties with emotion regulation have been consistently linked to suicidal thoughts (albeit less robustly with behaviors), and deficits in this skillset are thought to explain why psychological pain sometimes leads to suicide and self‐injury (McHugh et al. 2019; Rogante et al. 2024; Shelef et al. 2015; Turton et al. 2021). The connection between impulsivity and suicide is less clear; however, specific dimensions of impulsivity are broadly associated with STBs (i.e., lack of premeditation, negative urgency) and thought to influence transitions from suicidal thoughts to behaviors (Anestis et al. 2014; Auerbach, Stewart, and Johnson 2017; Beach et al. 2022; Bruno et al. 2023; Klonsky and May 2010). Although emotion dysregulation and impulsivity seldom predict suicide‐related outcomes with a high degree of specificity, they are thought to contribute to STBs through transactions with intrapersonal (e.g., biology) and interpersonal (e.g., relationship contexts) variables (van Heeringen 2012).

Taken together, research suggests that identity pathology and emptiness are independently associated with STBs. Literature also highlights the potential role of impulsivity and emotion dysregulation in understanding how these experiences may confer risk for SI. A formal test of potential risk pathways may advance our understanding of suicide risk, enable researchers to refine existing frameworks of psychopathology, and generate new hypotheses about these understudied correlates. Although research on *how* identity pathology and emptiness potentiate SI is sparse, there is evidence to suggest that identity pathology is indirectly related to STB via other variables (e.g., relationship dysfunction; Ren et al. 2018). Accordingly, in line with prior research, we examined whether emptiness mediates the link between identity pathology and SI. We tested whether emotion dysregulation and impulsivity interact with these processes to exacerbate the risk of SI. Specifically, this study evaluates the following hypotheses. First, we hypothesized that identity pathology would be directly associated with emptiness and SI, and indirectly associated with SI via emptiness. Second, we hypothesized that the relation between emptiness and SI would be stronger for those experiencing high (vs. low) levels of emotion dysregulation and impulsivity, controlling for baseline SI and life purpose.

## 1 Method

### 1.1 Participants

The sample comprised 251 university students aged 17 to 52 (*M* age = 21.67 years, SD = 6.97)^1^ recruited from (institution removed for review) via research subject pool and online and in‐person advertisements.^2^ We oversampled for persons with a history of STBs by recruiting those in the top one‐third of the distribution of scores on screening questionnaires (see [OSF link removed for review] for more details). The sample was largely female (*n* = 210, 83.7%), heterosexual (*n* = 174, 69.32%), and White (*n* = 175, 69.72%). The sample largely consisted of undergraduate students (*n* = 234, 93.22%) with a lifetime history of SI (*n* = 183, 72.9%). The most commonly reported suicidal thought was passive: “I wish I could disappear or not exist” (*n* = 56, 22.3%). In the week prior to participation, 46 individuals experienced SI. Demographic details and clinical characteristics are listed in Tables 1 and 2, respectively.

**TABLE 1:** Demographic characteristics of the sample.

	*N* (%)
Gender	
Female	210 (83.67)
Male	31 (12.35)
Non‐binary, genderqueer, or two‐spirit	5 (2.00)
Prefer not to say	1 (0.41)
Sexual Orientation	
Heterosexual	174 (69.32)
Bisexual	50 (19.92)
Gay or Lesbian	11 (4.38)
Asexual	1 (0.41)
Pansexual	3 (1.20)
Queer	3 (1.20)
Demisexual	1 (0.41)
Other	1 (0.41)
Race	
White	175 (69.72)
Asian	67 (26.69)
Black	13 (5.18)
Indigenous	7 (2.79)
Mixed Race	3 (1.20)
Hawaiian/PI	—
Ethnicity	
Hispanic	8 (3.19)
Middle Eastern	7 (2.79)
Occupational Status	
Student	179 (71.31)
Unemployed	85 (33.86)
Employed	82 (32.67)
Retired	2 (0.08)
Other	5 (2.0)
Current Education Level	
Undergraduate student	234 (93.22)
Professional student	7 (2.79)
Graduate student	4 (1.59)

*Note:* The “Other” response for sexual orientation was specified as “any or whatever” category.

Abbreviation: PI, Pacific Islander.

**TABLE 2:** Clinical characteristics of the sample.

	*N* (%)	*M* (SD)
Lifetime SI	183 (72.91)	
Lifetime SP	135 (53.78)	
Lifetime S Prep	78 (3.11)	
Lifetime SA	61 (24.30)	
Age of first SA		15.33 (3.67)
Lifetime NSSI	137 (54.58)	
Age of first NSSI		14.27 (3.30)
Past year NSSI (Num)		10.71 (24.66)

*Note:* Labels: Lifetime reflects a behavior or experience present at any point in one's life; (Num) denotes the number of times participants engaged in each behavior.

Abbreviations: NSSI, non‐suicidal self‐injury; S Prep, preparatory actions taken for a suicide attempt; SA, suicide attempt; SI, suicidal ideation; SP, planning (i.e., thinking of a way or method of suicide attempt).

### 1.2 Measures

Demographic and clinical details were assessed using a self‐report survey administered electronically via Qualtrics at baseline and at 2‐month follow‐up. Participants completed the study remotely.

#### 1.2.1 Demographic Questionnaire

Participants answered survey questions about the following demographic characteristics: age, sex assigned at birth, gender, sexual orientation, education level, occupational status, and ethnic identity.

##### 1.2.1.1 Zanarini Rating Scale for Borderline Personality Disorder

The Zanarini rating scale for borderline personality disorder (ZAN‐BPD) is a self‐report instrument assessing the frequency of each of the nine symptoms of BPD: anger, unstable mood, emptiness, identity pathology, self‐injury/STB, impulsivity, paranoid ideation, efforts to avoid abandonment, and unstable relationships. Each is scored from 1 (*I do not experience this*) to 5 (*I experience this every day*). Higher scores reflect more frequent symptoms. A total score is generated by summing each of the nine subscale scores. The ZAN‐BPD is shown to have high convergent validity (i.e., with clinical interview measures), good internal consistency, and excellent test–retest reliability (Zanarini et al. 2003; Guo et al. 2021; Zanarini et al. 2015).

##### 1.2.1.2 Depressive Symptom Index‐Suicidality Subscale

The depressive symptom index‐suicidality subscale (DSI‐SS) is a brief 4‐item self‐report measure of SI risk occurring over the past 2 weeks. Items are rated on a 4‐point scale from 0 (*I do not have these thoughts*) to 3 (*I always have these thoughts*). Responses are summed such that higher scores reflect more severe SI. In the current study, SI was assessed at baseline and 2 months later (McDonald's omega, *ω* = 0.92)^3^. The DSI‐SS has been validated in samples ranging in clinical severity and differentiates well between those with and without a past suicide attempt (von Glischinski et al. 2016; Stanley et al. 2021). The DSI‐SS is a psychometrically sound measure of SI risk, with evidence of excellent internal consistency, convergent and discriminant validity, sensitivity to change, and measurement invariance across gender, sexual orientation, race, ethnicity, and age (Batterham et al. 2015; Jeon et al. 2024; Joiner Jr, Pfaff, and Acres 2002; Stanley et al. 2021).

##### 1.2.1.3 Difficulties in Emotion Regulation Scale

The difficulties in emotion regulation scale (DERS‐18) is an 18‐item, brief version of the Difficulties with Emotion Regulation Scale (DERS; Gratz and Roemer 2004). This self‐report instrument assesses individuals' perceived capacity to regulate their emotions across 6 subscales: Awareness (lack of awareness of one's emotions), Clarity (poor clarity about the nature of one's emotions), Non‐acceptance (lack of acceptance of one's emotions), Strategies (lack of access to effective emotion regulation strategies), Goals (lack activities during negative emotions), and Impulse (lack of ability to manage one's impulses during negative emotions). Responses are scored from 1 (*almost never*) to 5 (*almost always*). After items are reverse‐scored, a score is calculated for each subscale and the overall measure. The internal consistency of the DERS‐18 full scale was excellent (*ω* = 0.92; see Table 3). The DERS‐18 has strong evidence of construct validity, internal consistency, and test–retest reliability in clinical and non‐clinical samples (Hallion et al. 2018; Victor and Klonsky 2016).

**TABLE 3:** Descriptive statistics and bivariate correlations.

	*n*	*M* (SD)	DSI‐SS	ZAN‐I	ZAN‐E	*ω*
DSI‐SS Baseline	251	1.14 (1.91)	0.64**	0.39**	0.37**	0.92
DSI‐SS Time 2	251	1.08 (1.90)	—	0.38**	0.43**	0.92
ZAN‐I	251	2.17 (1.22)	0.38**	—	0.55**	—
ZAN‐E	251	2.53 (1.34)	43**	0.55**	—	—
DERS‐18 Total	251	47.96 (14.29)	0.40**	0.57**	0.61**	0.92
DERS‐18 A	251	6.8 (2.71)	0.17**	0.20**	0.19**	0.79
DERS‐18 S	251	7.93 (3.55)	0.42**	0.50*	0.57**	0.85
DERS‐18 N	251	8.08 (3.83)	0.32**	0.44**	0.46**	0.91
DERS‐18 I	251	6.42 (3.48)	0.27**	0.38**	0.37**	0.93
DERS‐18 G	251	11.42 (3.38)	0.23**	0.42**	0.45**	0.93
DERS‐18 C	251	7.30 (2.91)	0.34**	0.52**	0.55**	0.85
UPPS‐P Total	245	42.70 (8.54)	0.31**	0.40**	0.44**	0.79
UPPS‐P PU	248	7.51 (2.80)	0.23**	0.32**	0.31**	0.80
UPPS‐P NU	247	9.73 (2.87)	0.36**	0.41**	0.42**	0.76
UPPS‐P S	245	10.07 (2.91)	0.01	0.08	0.18**	0.67
UPPS‐P P	251	7.71 (2.48)	0.24**	0.20**	0.22**	0.74
UPPS‐P Pre	245	7.71 (2.63)	0.18**	0.28**	0.25**	0.83

*Note: ω* = McDonald's omega coefficient for internal consistency.

Abbreviations: DERS‐18, Difficulties in Emotion Regulation Scale 18; DSI‐SS Baseline, baseline SI; DSI‐SS Time 2, 2‐month follow‐up SI; DSI‐SS, Depressive Symptom Index‐Suicidality Subscale; ZAN‐E, ZAN‐BPD emptiness; ZAN‐I, ZAN‐BPD identity pathology; Subscales: A, emotional awareness; C, emotional clarity; G, goal‐directed behavior; I, impulse control; N, nonacceptance of emotional responses; S, emotion regulation strategies; UPPS‐P, Impulsive Behavior Scale; Subscales: NU, negative urgency; P, perseverance; Pre, premeditation; PU, positive urgency; S, sensation‐seeking.

* *p* < 0.05, **p < 0.001.

##### 1.2.1.4 UPPS‐P Impulsive Behavior Scale

The UPPS‐P is a 20‐item measure assessing impulsivity severity (i.e., acts, thoughts, and urges). Responses are scored on a scale from 1 (*strongly agree*) to 4 (*strongly disagree*) across 5 subscales: Sensation Seeking (tendency to pursue novel and thrilling experiences), Premeditation (tendency to not consider consequences of actions), Perseverance (tendency to have difficulty focusing on challenging or mundane tasks), Negative Urgency (the tendency to act rashly when experiencing negative affect), and Positive Urgency (the tendency to act rashly when experience positive affect). A total score is calculated by summing each subscale score. The internal consistency of the full scale was good (*ω* = 0.79). The UPPS‐P shows consistently high internal consistency, test–retest reliability, and construct validity across studies, and is invariant across gender and age (Billieux et al. 2012; Dugré et al. 2019; Pompeia et al. 2018).

##### 1.2.1.5 Self‐Injurious Thoughts and Behaviors Interview—Revised

The self‐injurious thoughts and behaviors interview—revised (SITBI‐R) is administered as a questionnaire assessing participants' methods, frequency, and other characteristics of self‐injurious thoughts and behaviors (SITBI; Nock et al. 2007). The SITBI‐R was administered at baseline and used to characterize the sample in terms of the lifetime history of STB. The SITBI‐R has excellent inter‐rater reliability and construct validity in adult and adolescent samples (Fox et al. 2020; Gratch et al. 2022).

##### 1.2.1.6 Patient‐Reported‐Outcomes Measurement Information System: Meaning and Purpose (PROMIS‐MP)

The PROMIS‐MP is a four‐item questionnaire assessing one's sense of having a life purpose, meaning, and reasons for living. Responses are scored on a scale from 1 (*not at all*) to 5 (*very much*) and summed to create a total score. This variable was included as a covariate. The internal consistency for the full scale in the current sample was good (*ω* = 0.89). The PROMIS subscales have strong construct and predictive validity and good internal consistency in clinical and nonclinical samples (Salsman et al. 2020; Broderick et al. 2013; Quach et al. 2016).

### 1.3 Procedure

Participants were recruited from the community and university research participant pools. Participants completed several screening questionnaires, including items from the SITBI‐R (Fox et al. 2020) and other measures assessing correlates of self‐injury. We also used targeted flyers and social media advertisements to recruit university students outside of the participant pool. Participants who expressed interest in the study were sent the consent form and a battery of questionnaires via Qualtrics. Contact information was collected for all participants in accordance with safety procedures. Participants were invited to complete a series of follow‐up surveys 2 months later, which included the DSI‐SS. Participants were compensated with either academic course credits or Amazon e‐gift cards.

### 1.4 Analytic Plan

Descriptive statistics and correlations between all study variables were calculated using IBM SPSS Statistics (Version 27). We then sequentially tested mediation and moderated mediation models with the PROCESS macro (Hayes 2013, 2018), using models 4 and 14, respectively. We used a 95% bias‐corrected bootstrap model with 5000 iterations, yielding confidence intervals that were used to infer the significance of model effects (i.e., interval ranges not including zero are indicative of two‐tailed *p* values < 0.05). We first tested a simple mediation model in which identity pathology is indirectly associated with SI (assessed at the 2‐month follow‐up) through emptiness. If significant, we tested for moderated mediation, with impulsivity and emotion dysregulation included as moderators of the relation between emptiness and SI. We entered baseline SI to test whether our models predict change in SI from baseline to 2 months later. We also controlled for life purposes given that it is a protective factor for suicide and relevant to identity pathology and emptiness (Kleiman and Beaver 2013).

## 2 Results

### 2.1 Preliminary Analyses

Descriptive statistics and bivariate correlations between study variables are summarized in Table 1. On average, participants fell between the moderate to serious range for identity pathology (*M* = 2.17, SD = 1.22) and emptiness (*M* = 2.53, SD = 1.34)^4^. With the exception of affective instability, emptiness, and identity pathology were the two most frequently reported BPD features in this sample. SI scores were in the low severity range on average (baseline SI: *M* = 1.14, SD = 1.91; follow‐up SI: *M* = 1.08, SD = 1.90). More than half the sample reported a lifetime history of SI (72.91%) or suicide planning (53.78%) and approximately 24% of the sample reported a lifetime history of suicide attempt.

### 2.2 Indirect Effects of Emptiness on Identity Pathology and SI

**FIGURE 1:**
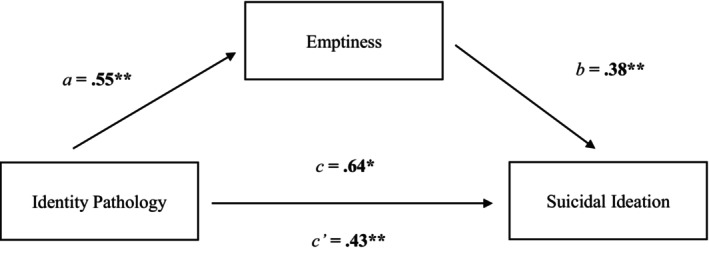
Mediation model. *Note*. Path values represent standardized coefficients for the mediation model. Identity pathology was indirect associated with SI via emptiness (*β* = 0.15, SE = 0.05, Bootstrap 95% CI = [0.06, 0.24]). *p < 0.05, **p < 0.001.

The results for the mediation model were consistent with our hypotheses. We found a positive association between identity pathology and emptiness assessed concurrently (*β* = 0.55, SE = 0.58, *p* < 0.001), and between emptiness and prospective SI (i.e., 2 months later; *β* = 0.38, SE = 0.10, *p* < 0.001). There was also a significant direct effect between identity pathology and SI (*β* = 0.43, SE = 0.11, *p* < 0.001), suggesting those with more severe identity pathology at baseline experienced elevated SI 2 months later. We found a significant indirect effect of identity pathology on SI through emptiness (*β* = 0.15, SE = 0.05, Bootstrap 95% CI = [0.06, 0.24]). Identity pathology was prospectively associated with SI, both directly and via experiences of emptiness (see Figure 1). When entering baseline SI and life purpose as covariates, both identity pathology (*β* = 0.13, SE = 0.09, *p* = 0.03) and emptiness (*β* = 0.15, SE = 0.08, *p* = 0.01) remained significant predictors of SI two months later. The effects of the mediation model were also significant (*β* = 0.07, SE = 0.03, Bootstrap 95% CI = [0.01, 0.14]).^5^

### 2.3 Moderated Mediation

Contrary to hypotheses, we did not observe interaction effects for emotion dysregulation (*β* = 0.01, SE = 0.10, *p* = 0.39) and impulsivity (*β* = 0.01, SE = 0.19, *p* = 0.19) total scores, nor did we observe moderated mediation effects.

**FIGURE 2:**
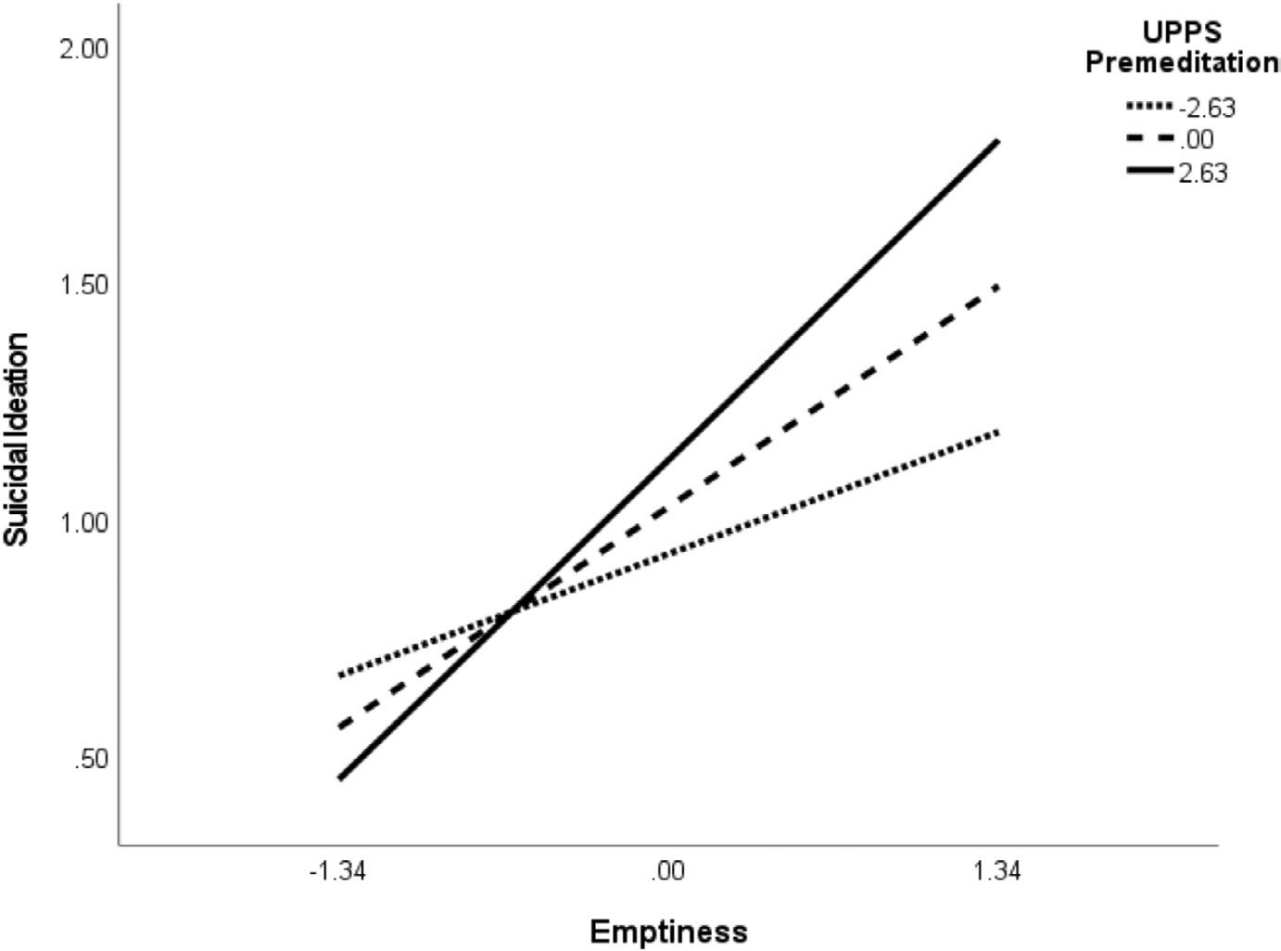
Suicidal ideation as a function of emptiness and impulsive lack of premeditation. *Note*. Emptiness and UPPS‐Premeditation were mean‐centered. Values of the moderator are −1 SD = −2.63 *M* = 0.00, +1 SD = 2.63. The relation between UPPS‐Premeditation and emptiness was statistically significant at all levels of the moderator, with the exception of 1 SD below the mean. Higher scores index greater impulsive lack of premeditation (i.e., greater impulsivity).

**FIGURE 3:**
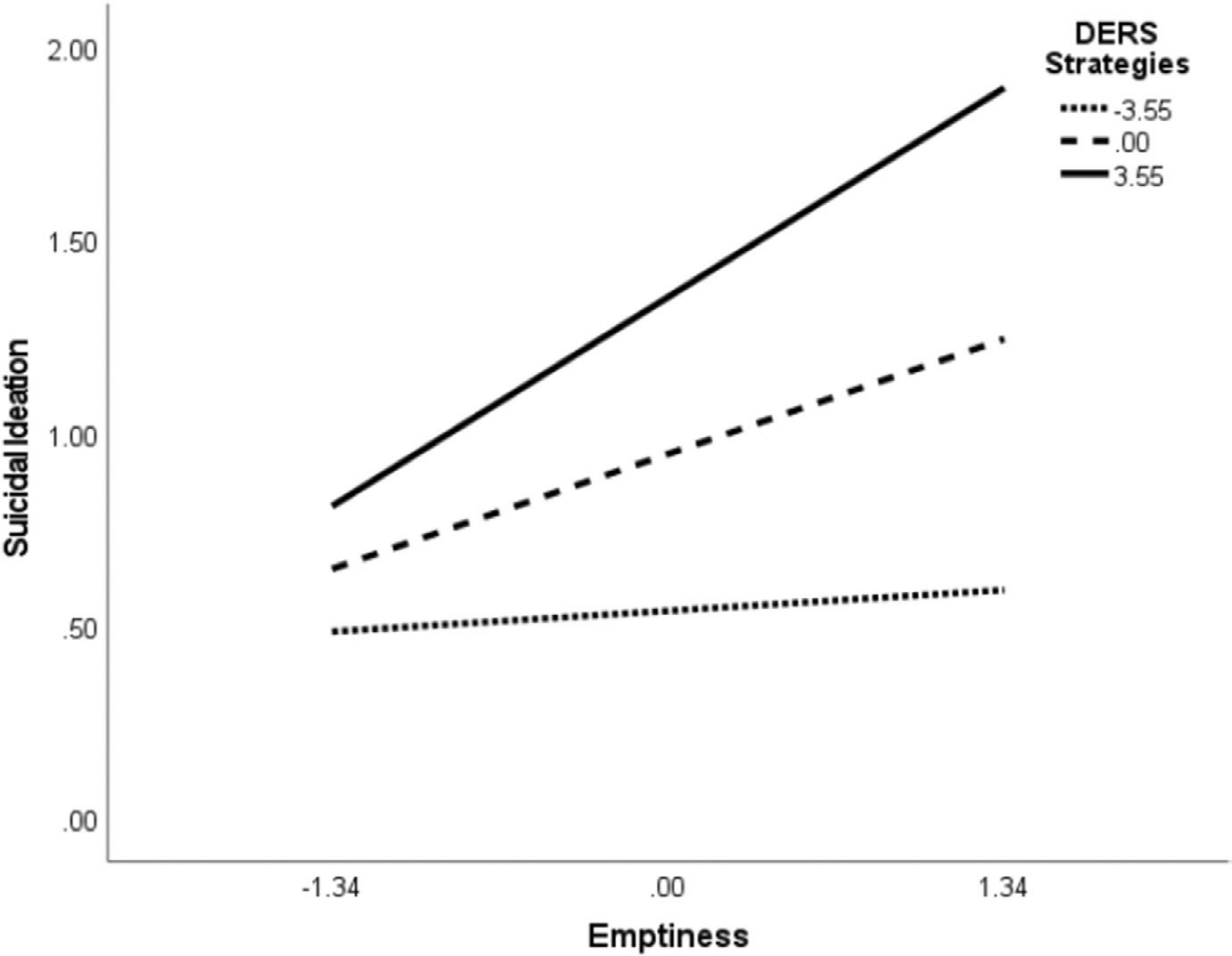
Suicidal ideation as a function of emptiness and emotion regulation strategies. *Note*. Emptiness and DERS‐Strategies were mean‐centered. Values of the moderator are −1 SD = −3.54, *M* = 0.00, +1 SD = 3.54. The relation between DERS‐Strategies and emptiness was statistically significant at all levels of the moderator, with the exception of 1 SD below the mean. Higher scores index greater perceived inability to use emotion regulation strategies. When controlling for baseline SI, the direct, indirect, and moderator effects of emotion regulation strategy use disappear.

**FIGURE 4:**
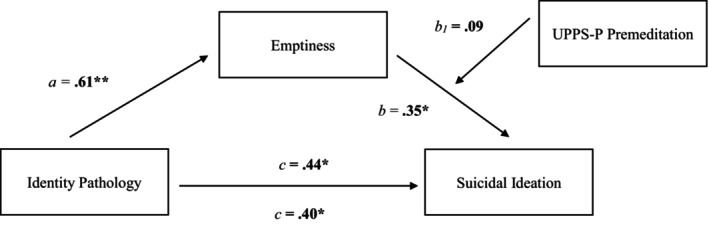
Moderated mediation model with lack of premeditation as moderator. *Note*. Path values represent unstandardized coefficients for the moderated mediation model. The index of moderated mediation was not significant (*β* = 0.09, *SE* = 0.03, Bootstrap 95% CI = [−0.01, 0.08]). *p* < 0.05, *p < 0.01, **p < 0.001.

**FIGURE 5:**
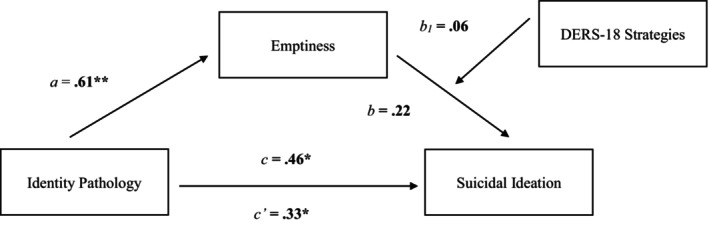
Moderated mediation model with lack of emotion regulation strategies as moderator. *Note*. Path values represent unstandardized coefficients for the moderated mediation model. The index of moderated mediation was not significant (*β* = 0.03, SE = 0.02, Bootstrap 95% CI = [−0.01, 0.07]). *p* < 0.05, *p < 0.01, **p < 0.001.

To further explore whether potential facets of emotion dysregulation and impulsivity interact with emptiness, we tested additional models parsing out subscales of the UPPS‐P and DERS‐18. We found evidence of two‐way interactions between emptiness and both the UPPS‐P premeditation (*β* = 0.09, SE = 0.03, *p* = 0.004) and DERS‐18 strategies (*β* = 0.06, SE = 0.03, *p* = 0.02) subscales predicting SI. Simple slope tests revealed that emptiness had a significant influence on SI at the mean (*β* = 0.37, SE = 0.10, Bootstrap 95% CI = [0.18, 0.57]) and 1 SD above‐the‐mean (*β* = 0.60, SE = 0.12, Bootstrap 95% CI = [0.36, 0.82]) values of UPPS‐P premeditation (Figure 2). Similar effects were observed for mean (*β* = 0.24, SE = 0.10, Bootstrap 95% CI = [0.04, 0.45]) and 1 SD above‐the‐mean (*β* = 0.44, SE = 0.12, Bootstrap 95% CI = [0.19, 0.67]) DERS‐18 strategies, with stronger effects observed at higher levels (see Figure 3). These conditional effects were not significant at low (−1 SD mean) levels of each moderator. After controlling for baseline SI, only interaction effects between emptiness and impulsive lack of premeditation remained significant at 1SD above the mean (*β* = 0.43, SE = 0.10, Bootstrap 95% CI = [0.10, 0.51]). The index of moderated mediation was not significant for either model. Both models with the standardized path estimates are displayed in Figures 4 and 5.

## 3 Discussion

Our results support the growing body of research suggesting identity pathology and emptiness are important indicators of SI risk. Identity pathology and emptiness were prospectively associated with SI, even after controlling for baseline SI. Further, in line with our hypotheses, the effect of identity pathology on future SI was partially mediated by emptiness. That is, those who reported greater uncertainty about who they are were more likely to experience emptiness, and this in turn was associated with more severe future SI. This finding aligns with BPD theories, which hypothesize that emptiness and identity problems are closely related and potentiate risk for STB (Kernberg 1967; Linehan 1993). In particular, we provide preliminary support for the hypothesis that emptiness plays a mechanistic role in conferring risk for STBs among those who struggle with their identity. This indirect effect remained significant controlling for baseline SI and life purpose. Thus, identity pathology is indirectly associated with *change in* SI via emptiness and this effect is unique beyond having a weak sense of purpose. Since the direct effects of identity pathology on SI remained significant, emptiness appears to be one mechanism by which identity pathology may confer risk for SI, at least for some people.

Although our findings do not speak directly to developmental effects, they support the view that identity problems accompany, and possibly beget, feelings of emptiness. When occurring together, both appear to elevate persons' suicide risk. Although we oversampled for STBs, participants reported moderately severe BPD symptoms on average, with most falling outside of the range that typifies BPD patients in clinical settings. Thus, identity pathology and emptiness remain meaningfully related to important clinical outcomes like SI, even for people without a BPD diagnosis. This is important, as few studies examine these transdiagnostic constructs outside of BPD (Kaufman, Cundiff, and Crowell 2015; Price et al. 2020). Even among persons with BPD, identity pathology and emptiness are likely two poorly understood mechanisms for suicide risk that could otherwise be an important focus of intervention work.

The moderating effects of overall emotion dysregulation and impulsivity were nonsignificant. This result is not entirely surprising, as the literature is mixed regarding which dimensions of the UPPS‐P and DERS‐18 are primarily implicated in suicide risk. For example, although impulsivity is generally higher among persons experiencing SI, effects are inconsistent across subscales (e.g., Anestis et al. 2011; Johnson et al. 2017). Researchers suggest that dimensions of emotion regulation may relate differently to suicidal thoughts versus behaviors (Law, Khazem, and Anestis 2015). In keeping with our findings, the DERS‐18 strategies subscale appears to be most strongly associated with SI across several studies (Rajappa, Gallagher, and Miranda 2012; Weinberg and Klonsky 2009).

Consistent with prior research, participants' perceived inability to use emotion regulation strategies appeared important for predicting SI (Weinberg and Klonsky 2009). Those who experience emptiness *and* perceive that they cannot use emotion regulation strategies may be more likely to experience SI. We observed a similar moderating effect for persons' lack of premeditation: participants who felt empty were more likely to experience SI when they also reported a tendency to act without considering the consequences. These effects were nonsignificant at low levels of both moderators, suggesting that emptiness no longer predicted SI when individuals reported better premeditation and perceived emotion regulation strategy use. Thus, deficits in these areas of functioning appear to have significant consequences for those who experience emptiness.

Emptiness is conceptualized as a subjective *lack* of emotional experience. However, researchers have not investigated how much emptiness reflects or relates to underlying deficits in emotion regulation and impulse control. Our preliminary findings suggest that poor perceived use of emotion regulation strategies may exacerbate distress associated with emptiness, as this subscale captures a more hopeless outlook on managing emotions (Gratz and Roemer 2004). Alternatively, individuals may believe they lack strategies to manage *emptiness*, specifically. Indeed, emptiness is related to affective states characterized by negative valence and low arousal (Klonsky 2008). Thus, it seems that emptiness may in and of itself evoke negative affective experiences (i.e., distress) requiring the use of emotion regulation strategies.

A lack of premeditation—defined as a diminished capacity to consider the consequences of one's actions—was the only impulsivity dimension that distinguishes suicide attempters from ideators in prior well‐designed studies (Klonsky and May 2010; Yen et al. 2009). This aligns with our findings as lack of premeditation was the only impulsivity dimension that emerged as a significant moderator. Since emptiness conceptually overlaps with boredom and disconnection, it is not surprising that the sensation‐seeking subscale was nonsignificant (Miller et al. 2020). Likewise, positive and negative urgency may be less applicable to emptiness given that urgency implies strong positive and negative emotions, whereas emptiness suggests a distressing *lack of* emotion.

Some of our moderated mediation results were nonsignificant after adjusting for baseline SI. Thus, these models were capturing variance in SI that was attributable to stable aspects of suicide risk, rather than changes in risk over the 2‐month follow‐up period. It is therefore unclear how emotion regulation strategy interacts with emptiness to elevate the *future* risk of suicide. In contrast, a greater impulsive lack of premeditation interacts with emptiness to predict increases in SI 2 months later. To prevent escalations in suicide risk, this facet of impulsivity may be especially important to target among those who feel empty.

## 4 Limitations and Future Directions

Our findings are preliminary and must be interpreted in light of several important limitations. First, our measures of identity pathology and emptiness were single items from a self‐report measure of BPD. However, unlike most prior research, we used items that were scored dimensionally, which may be a methodological advantage of this work. Nevertheless, our assessments may have had insufficient domain coverage, and our findings only apply to individuals with a restricted range of construct‐relevant experiences. This is an important limitation and underscores the need for future research to extend and replicate these findings using comprehensive transdiagnostic measures (i.e., The Self‐Concept and Identity Measure, Kaufman, Cundiff, and Crowell 2015; The Subjective Emptiness Scale, Price et al. 2020). We were unable to perform a rigorous test of mediation (i.e., in which the mediator emerges as a result of the predictor), as emptiness was assessed concurrently with identity pathology (Judd and Kenny 1981; Cole and Maxwell 2003). Future research is therefore needed to clarify the relation between these experiences across development. Using longitudinal methods, researchers should investigate causal pathways to delineate how these phenomena transact over time to predict poor outcomes. This is especially pertinent, as these vulnerabilities are salient during specific developmental periods (e.g., identity confusion is normative during adolescence; Lutz and Ross 2003).

Since our sample was composed largely of White, heterosexual, and cisgender women, our findings may not generalize to more representative groups and need to be replicated in demographically diverse samples. Indeed, suicide disproportionately affects persons from marginalized backgrounds and key risk factors have a greater impact on certain vulnerable groups (Bostwick et al. 2014; Hottes et al. 2016). Identity pathology may also be especially relevant for those who experience greater social barriers to identity development or face discrimination for facets of their identities (e.g., gender and racial; Jackson et al. 2012). The literature would benefit from studies that integrate and test theories of identity pathology, suicide, and minority identity development and stress.

Future studies should also clarify how and to what extent emotion dysregulation and impulsivity are important for understanding identity pathology and emptiness. Other potential mediators and moderators may shed light on *how* and *for whom* these experiences are especially harmful. For example, some studies suggest that emptiness commonly co‐occurs with feelings of hopelessness and isolation among those vulnerable to STB (Klonsky 2008; Miller, Townsend, and Grenyer 2021). Interpersonal difficulties may play a particularly important role in predicting STBs in persons with identity problems, as identity is shaped through social processes (e.g., feedback; Kaufman and Crowell 2018; Lutz and Ross 2003). Given that identity pathology was directly and indirectly associated with SI in this study, it will be important to assess whether certain facets of this experience are better explained by emptiness. Perhaps emptiness is a stronger mechanism for SI among those who perceive a *lack of identity*—as both experiences capture a sense of lacking/wanting (see Kaufman, Cundiff, and Crowell 2015). Further, these phenomena likely have behavioral and biological markers of risk, and possibly similar risk indicators as emotion dysregulation and impulsivity (Kaufman and Crowell 2018; Linehan 1993). Multimethod assessment may allow for the detection of vulnerabilities that can strengthen predictive models and elucidate risk mechanisms. Finally, future studies should examine these experiences among clinical samples to predict suicide‐related outcomes with greater specificity and uncover potential unique trajectories of risk (e.g., emptiness and identity problems may present, and function, differently in BPD versus depression; Miller et al. 2020). Doing so could help elucidate the potential clinical utility of assessing identity pathology and emptiness in various settings (e.g., as a part of suicide risk screening or marker of treatment progress). Likewise, it is unknown whether and to what extent existing psychological interventions can address issues related to identity and emptiness.

This study helps to build an empirical foundation for neglected, but important, clinical constructs. Our preliminary findings add to the burgeoning literature highlighting identity pathology and emptiness as important predictors of STB. These experiences may also serve as promising targets for suicide intervention—particularly if they can be addressed concomitantly or using existing treatments (e.g., Dialectical Behavior Therapy or Acceptance and Commitment Therapy, Linehan 1993; Hayes, Strosahl, and Wilson 1999). Addressing persons' difficulties using emotion regulation strategies and increasing premeditation may help protect against the adverse effects of emptiness on SI. In sum, our study represents an important step in this line of research, yet more attention should be directed to investigating how identity pathology and emptiness increase suicide risk.

## Ethics Statement

This study was approved by the institutional review board at Queen's University (REB #GPSYC‐990‐20).

## Conflicts of Interest

The authors declare no conflicts of interest.

## Data Availability

Data and materials are available upon request.

## Endnotes

^1^
Age data were missing for *n* = 6 participants.

^2^
Complete baseline and follow‐up data were available for all participants for the initial mediation model; however, 6 participants were excluded from moderated mediation analyses due to missing data on the emotion regulation or impulsivity measures.

^3^
The DSI‐SS showed moderate test–retest reliability (ICC = 0.66, 95% CI = [0.58, 0.73]) across the two administrations, which is typical of many SI measures. Some inconsistency is expected in this reliability index given that suicidal thoughts are variable over time.

^4^
Scores range from 0 to 5 (no symptoms to severe symptoms).

^5^
In the overall model, life purpose was not a significant predictor of SI (*β* = − 0.10, *SE* = 0.04, *p* = 0.50).
